# Description and characterization of a novel live-attenuated tri-segmented Machupo virus in Guinea pigs

**DOI:** 10.1186/s12985-018-1009-4

**Published:** 2018-06-07

**Authors:** Amélie D. Zaza, Cécile H. Herbreteau, Christophe N. Peyrefitte

**Affiliations:** 1 Fab’entech, 24 rue Jean Baldassini Bat B 69007, Lyon, France; 2 Unité de virologie, Institut de Recherche Biomédicale des Armées, 1 place Valérie André, 91220 Brétigny-sur-Orge, France; 3 UMR 190, Faculté de Médecine-Timone, 27 boulevard Jean-Moulin, 13385 Marseille, France

**Keywords:** Vaccine candidate development, Therapeutic, Mammarenaviruses, Machupo virus, Junín virus, Candid #1, Tri-segmented virus, Reverse genetic approaches

## Abstract

**Background:**

Machupo virus (MACV) is a member of the *Mammarenavirus* genus, *Arenaviridae* family and is the etiologic agent of Bolivian hemorrhagic fever, which causes small outbreaks or sporadic cases. Several other arenaviruses in South America Junín virus (JUNV) in Argentina, Guanarito in Venezuela, Sabiá in Brazil and Chapare in Bolivia, also are responsible for human hemorrhagic fevers. Among these arenaviruses, JUNV caused thousands of human cases until 1991, when the live attenuated Candid #1 vaccine, was used. Other than Candid #1 vaccine, few other therapeutic or prophylactic treatments exist. Therefore, new strategies for production of safe countermeasures with broad spectrum activity are needed.

**Findings:**

We tested a tri-segmented MACV, a potential vaccine candidate with several mutations, (r3MACV). In cell culture, r3MACV showed a 2-log reduction in infectious virus particle production and the MACV inhibition of INF-1β was removed from the construct and produced by infected cells. Furthermore, in an animal experiment, r3MACV was able to protect 50% of guinea pigs from a simultaneous lethal JUNV challenge. Protected animals didn’t display clinical symptoms nor were virus particles found in peripheral blood (day 14) or in organs (day 28 post-inoculation). The r3MACV provided a higher protection than the Candid #1 vaccine.

**Conclusions:**

The r3MACV provides a potential countermeasure against two South America arenaviruses responsible of human hemorrhagic fever.

**Electronic supplementary material:**

The online version of this article (10.1186/s12985-018-1009-4) contains supplementary material, which is available to authorized users.

## Findings

Machupo virus (MACV) belongs to the *Mammarenavirus* genus of the *Arenaviridae* family and is the etiologic agent of Bolivian Hemorrhagic Fever (BHF) [1], discovered in 1959. From 1962 to 1964, it caused 515 human cases, 114 of which were fatal [1]. Then, 40 years later, from 2006through 2008, 200 human cases were reported including 12 deaths [2]. Similarly, the 1950s, another arenavirus, Junín virus (JUNV), was responsible for cases of human hemorrhagic fever (HHF) in Argentina [3]. Since 1991, the use of the Candid #1 vaccine, a live-attenuated vaccine against JUNV, decreased the case number [3]. Furthermore, three others mammarenaviruses have caused HHF outbreaks in South America and include Guanarito (GTOV), Chapare (CHAPV) and Sabiá (SABV) viruses [4–7]. Post-exposure passive transfer of human plasma-derived antibodies was used for humans and also non-human primates (NHPs) challenged by JUNV and MACV [3, 8]. However, this treatment induced a late-neurological syndrome [8]. Although Ribavirin reduced lethality in humans, administration at very early stage of infection was necessary [9] due to its side effects [3]. Therefore, alternative strategies with a broad spectrum activity are needed. Here, we report the use of the reverse genetic strategy to design a potential vaccine candidate targeting MACV and JUNV.

**Fig. 1:**
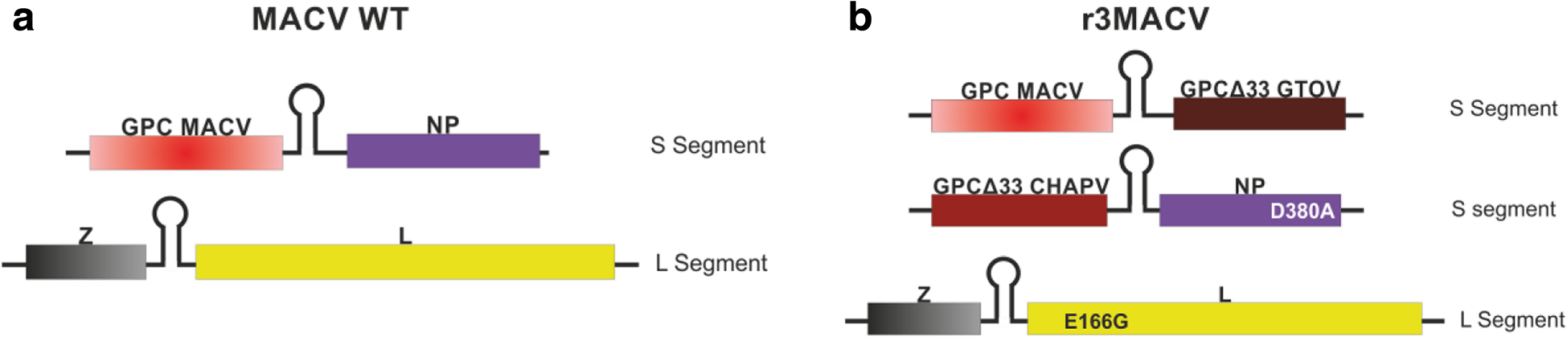
Schematic representation of MACV wt and r3MACV genomic constructs. Arenaviruses have a bisegmented negative single-stranded RNA genome. **a** For the MACV wt, the S segment encodes the viral glycoprotein precursor (GPC) and the nucleoprotein (NP), whereas L segment encodes the viral polymerase (L) and the small RING finger protein Z. **b** For the r3MACV, one S segment encodes the MACV GPC and the GTOV GPCΔ33, and the other encodes the CHAPV GPCΔ33 and the MACV NP, and the L segment is unchanged than the wt genome. Mutations inserted in r3MACV genome were noted

Mammarenaviruses are enveloped viruses with a single-stranded RNA genome composed of two segments [9]. Each segment encodes two ambisense genes, separated by a hairpin intergenic region (Fig. 1a). The L segment contains the genes of the small ring finger Z protein and of the RNA-dependent RNA polymerase L protein. In the S segment are found the genes of the viral nucleoprotein (NP) and of the glycoprotein complex (GPC). It was previously demonstrated that the bi-segmented genome of arenaviruses could be modified into a tri-segmented genome, by the duplication of the S segment [10]. To select and maintain tri-segmented viruses, the NP gene was removed from one of the two S segments, and the GPC gene was removed from the second S segment, so that two genes of interest could be inserted into these empty loci (Fig. 1b). Prior studies demonstrated efficacy using this approach for Old World Lymphocytic choriomeningitis virus (LCMV) or the New World JUNV [10, 11]. They constructed a tri-segmented JUNV (r3JUNV) or LCMV (r3LCMV), the latter of which showed high attenuation vs WT and exhibited protection against LCMV challenge when used as a vaccine candidate in mice [10].

In these studies, we used the reverse genetic system applied to the MACV Carvallo strain genome. As illustrated in Fig. 1 we generated three S segments, a wild-type one along with two genetically modified S segments, one harboring the CHAPV strain 810,419 GPC gene (acc # EU260463.1) into the MACV GPC gene locus, and the second displaying GTOV strain INH-95551 GPC gene (acc # AY129247.1) into the MACV NP gene locus. This virus was named r3MACV (Fig. 1b). Thirty three (33) amino acids were deleted from the C-termini of the two GPC sequences, required for production of infectious viral particles [12], and avoid any competition with the MACV wt GPC. Such a competition might have changed the virus cellular tropism or led to the loss of one of the two modified S segment. Additional modifications aimed to stimulate the adaptive immune response, by insertion of mutation (D380A in NP gene), shown to prevent the MACV NP IFN type-I inhibition [13]. Finally, glutamic acid was replaced by glycine at position166 (E166G) in the polymerase sequence. This mutation resulted in an 8-fold decrease in polymerase activity (mini-genome assay, Additional file 1). MACV and JUNV are closely genetically related, and, indeed, Candid #1, protected guinea pigs and primates from a lethal MACV challenge [14]. Since SABV and CHAPV are genomically closely related [6], r3MACVwas designed to target MACV, JUNV and each these additional South America arenaviruses responsible of HHF.

To replicate the pattern of ther3LCMV construct, reverse genetic systems were designed using synthesized Z, GPC, NP, NP D380A and L genes as well as S and L backbones (Eurofins Genomics, France) based on published sequences (GenBank acc # AY619643 and AY619642). The S and L backbones contained all the non-coding regions of the related viral segment, and the genes loci were replaced by BsmBI (GPC and L loci), BbsI (NP locus) or SapI (Z locus) restriction sites (Fig. 1). These sites were designed to further eliminate all the non-viral sequences after the enzymatic restriction step. Restriction sites required to clone the genes into the backbones or the pCAGGs plasmids were either included during the gene synthesis, or added by PCR using primers with 5′ extensions using the PCR Extender system (5PRIME) or the Q5 High-Fidelity PCR Kit (New England Biolabs), following manufacturer’s recommendations [10]. The E166G polymerase mutation was inserted into the expression plasmids using the Q5 site-directed mutagenesis kit (New England Biolabs). Amplified products were then purified using the QIAquick PCR Purification kit (Qiagen) and digested with the corresponding enzyme (BsmBI, BbsI or SapI, New England Biolabs). Restriction products were gel purified using agarose (Thermo Fisher) and the QIAquick Gel Extraction Kit (Qiagen) then ligated using the Quick Ligation Kit (New England Biolabs). Ligated products were transformed into the NEB 10-β or NEB 5-α F’Iq Competent *E. coli* (New England Biolabs). The bacterial clones were screened using the PCR (PCR Master Mix, Thermo Fisher) following the manufacturer’s instructions. The positive clones were grown in 50 ml LB broth (Thermo Fisher), then the plasmids were extracted using the Plasmid Plus Midi Kit (Qiagen). Two vectors, kindly provided by JC de la Torre, were used: the pCAGGs vector to express the viral proteins [15], and the pol I vector to produce the viral RNA segments [16]. The r3MACV genomic sequences are shown in Additional file 2.

**Fig. 2:**
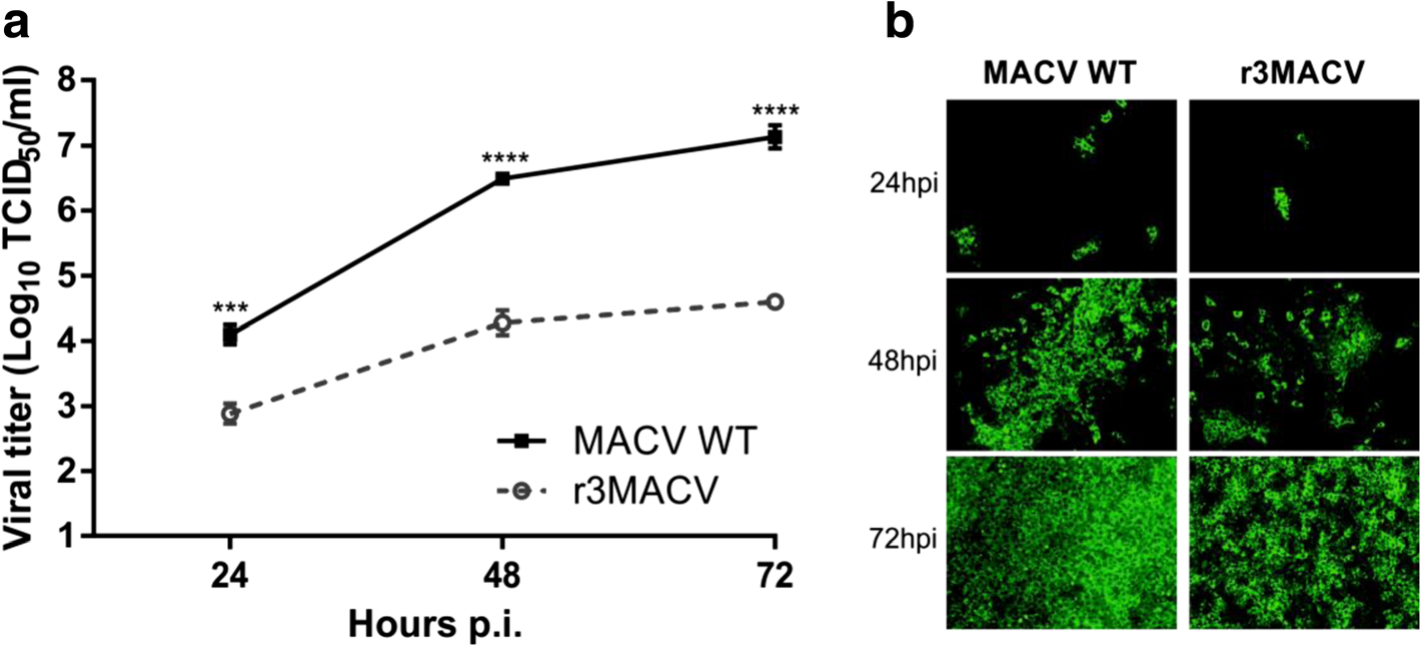
Growth properties of the r3MACV. VERO cells were infected for one hour with the MACV wt or the r3MACV viruses at a MOI of 0.01. The inoculum was then removed and with culture medium (DMEM GlutaMax 1% supplemented with 2% fetal bovine serum) was added. **a** At indicated time points, virus titers in the supernatant solutions were determined by virus TCID_50_ (50% tissue culture infective dose) using the end-point dilution assay and the Reed-Müench calculation method. Viral titers were determined in octuplicates using immunofluorescence [11] with the primary mouse monoclonal antibody anti-MACV NP. **b** At indicated time points, cells were fixed with PFA 4% (Electron microscopy sciences) and permeabilized with PBS containing 0.3% Triton X-100 (Merck) and 3% bovine serum albumin (Sigma). Viral NP expression was revealed using an Alexa Fluor 488 secondary antibody (Thermo Fisher). An epifluorescence optical microscope (× 100) was used for the eGFP observation. This experiment was done in triplicate. Asterisks denote significant differences (*P* < 0.05, two-way ANOVA with the Bonferroni correction, *** ≤ 0.001, **** ≤ 0.0001)

After the construction and production of the MACV wt and r3MACV, using conditions previously described [11], viruses were concentrated and purified using an ultracentrifugation step (28,000 rpm for 2 h at 4 °C in a Beckman sw32i rotor) on a sucrose (Life technologies) gradient (20 to 60%). Pellets were resuspended into PBS (Life technologies). We then compared their replication and propagation in VERO cells at a multiplicity of infection (MOI) of 0.01. We observed at 48 and 72 h post-infection (hpi), that r3MACV titers were lower (2 × 10^4^ and 4 × 10^4^ TCID_50_/ml) than the MACV wt titers (3 × 10^6^ and 1 × 10^7^ TCID_50_/ml) (Fig. 2a). We also noted that the r3MACV infected fewer Vero cells than the MACV wt (Fig. 2b). In conclusion, the r3MACV genomic modifications resulted in a decrease of the virus infectivity in Vero cells.

To test the genomic stability, an additional fifth passage of the r3MACV viral stock was produced using conditions previously described for the production [11]. Briefly, the supernatants from MOI 0,01 infected Vero cells were collected 3 days post infection and centrifuged 15 min, 1610 g, + 4 °C centrifuged before being passaged using these conditions. The viral stock and the late fifth passage were extracted (QIAmp Viral RNA Mini Kit, Qiagen) and sequenced. The experiments were done in triplicate. All results of r3MACV sequencing were similar. Sequencing revealed three changes between r3MACV and MACV wt in the L-segment. Two changes resulted in false sense mutation (T➔C) at position 1733 (leucine ➔ proline) and at position 6598 (serine ➔ proline) in the polymerase. We also identified a 35 nt deletion in the intergenic region (on position 417 to 452). Sequencing revealed one change under one S segment of r3MACV: surprisingly, we found an 1124 nt deletion in the C-termini of the CHAPV GPCΔ33. These deletions were observed in the GP1 and GP2 sequences of the GPC, which are involved in the cellular attachment and entry of the virion [9, 12]. Further experimentations will be needed to understand the consequences of these observations. However we can speculate that an excess of SSP-GP1 residual complex was expressed at the cell surface, moreover this truncated CHAPV GPC was likely not able to produce any infectious virions [12]. No genetic changes in the others genes were identified. In summary, r3MACV showed a moderate genetic stability after 5 passages, regarding the conservation of tri-segmented segments and the mutations inserted. However to support the safety of r3MACV, the deletion inside the CHAPV GPCΔ33 needed to be studied. The deletion of 35 nt in the L segment is interesting. As shown by Golden and his collaborators, a variant of this viral strain, also having a deletion of 35 nt but forward 8 nt (position 409–443) in the intergenic region of L segment, is not pathogenic for guinea pigs [17]. These data suggested a confidence in the safety of r3MACV. Consequently, the CHAPV GPCΔ33 representation in Fig. 1b was not entirely present in the r3MACV virus.

**Fig. 3:**
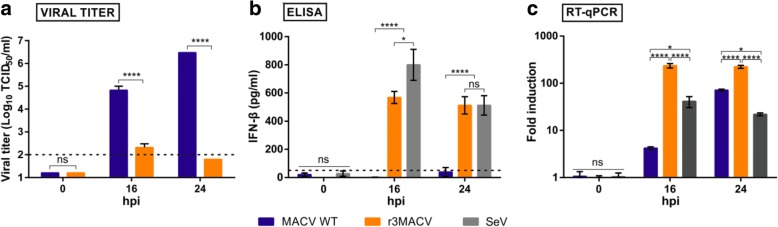
IFN-β production by A549 cells infected either with MACV wt, r3MACV or SeV. A549 cells were infected for one hour with the MACV wt, r3MACV or SeV viruses at a MOI of 3, then the inoculum was removed and fresh culture medium was added. At indicated time points, **a** virus titers in the supernatants were determined by virus TCID_50_, as described in Fig. 2; **b** production of IFN-β was detected in supernatant solutions using the VeriKine Human IFN BETA ELISA Kit (PBL assay sciences); and **c** intracellular RNA was extracted using the QIAmp Viral RNA Mini kit (Qiagen). RT-qPCR were performed using the QuantiTect Probe RT-PCR kit (Qiagen) in a 30 μl final volume with 5 μl of purified RNA and primers and probe at a final concentration of 400 nM and 200 nM, respectively. The assay was carried out using a CFX96 model (Bio-Rad) with a cycling profile of 50 °C for 30 min, 95 °C for 15 min, and 40 cycles at 94 °C for 15 s followed by 60°c for 1 min. The primers and probes (available on request) were designed using the Eurofins Genomics’ online tool (www.eurofinsgenomics.eu). Probes were 5′- and 3′-labelled with the fluorescent reporter dye 6-carboxyfluorescein (FAM) and the Black Hole Quencher (BHQ-1), respectively. All procedures were carried out following manufacturers’ instructions. This experiment was carried out in triplicate. Asterisks denote significant differences (P < 0.05, two-way ANOVA with the Bonferroni correction, ns: no significate, * ≤ 0.05, **** ≤ 0.0001)

To assess that the r3MACV NP D380A mutation removed the NP wt inhibition of the host type-I IFN response, we compared the A549 cells IFN-1β production in different conditions (Fig. 3): infection at a MOI of 3 by MACV wt, r3MACV and Sendaï virus (SeV) a known inducer of type-I IFN [18]. MACV wt was able to produce an infectious progeny (at 3 × 10^6^ TCID_50_/ml) up to 24 hpi (Fig. 3a) whereas the infectious particles produced by r3MACV were essentially undetectable (Fig. 3a). SeV replication induced the production of IFN-β at an average of 512 pg/ml (Fig. 3b), as previously reported. INF-1 β was undetectable in cultures of MACV wtat 16 and 24 hpi (Fig. 3b) while r3MACV, similarly to SeV, induced 527 pg/ml of IFN-β (average) at 24 hpi (Fig. 3b). We further confirmed this observation using RT-qPCR (Fig. 3c). Together, these findings demonstrated that r3MACV is no longer capable to block the IFN production from infected cells.

In order to analyze the protective capability of r3MACV, Dunkin-Hartley guinea pigs (females, 200–250 g, Charles River) animals were inoculated by intraperitoneal injections in a 0.5 ml PBS final volume in a biosafety level (BSL)-4 laboratory (Jean Merieux, Lyon, France). During all manipulation steps excluding weighing (3 times per week), guinea pigs were anesthetized by isoflurane 3% inhalation. Animals were humanly euthanized when end points were attained.

The MACV strain Carvallo pathogenicity has been demonstrated in guinea pigs (lethality ~ 60%) [19, 20]. To determine the wt MACV lethal dose in this experiment, two groups of guinea pigs were inoculated using 3 × 10^4^ TCID_50_ or 10^6^ TCID_50_. In the two conditions, animals survived, without losing weight and did not develop any HHF symptoms (see Additional file 3). This result indicated that, in our hands, MACV wt was not lethal despite the high doses tested, also a recent observation by other researchers [21]. Therefore, we then chose the Espindola strain of JUNV, closely genetically related to MACV, which was shown to induce 100% mortality in guinea pig, following weight loss and hemorrhagic symptoms [22]. We also selected the Candid #1 vaccine as comparison. Even if it requires 2 months following the vaccination to induce a protection against JUNV [14], we believed it would add meaningful information, as another live-attenuated virus. Following the JUNV challenge, we administered a low dose of r3MACV and Candid #1. However, the r3MACV dose was 3 times lower than expected because the r3MACV production in VERO cells was low.

**Fig. 4:**
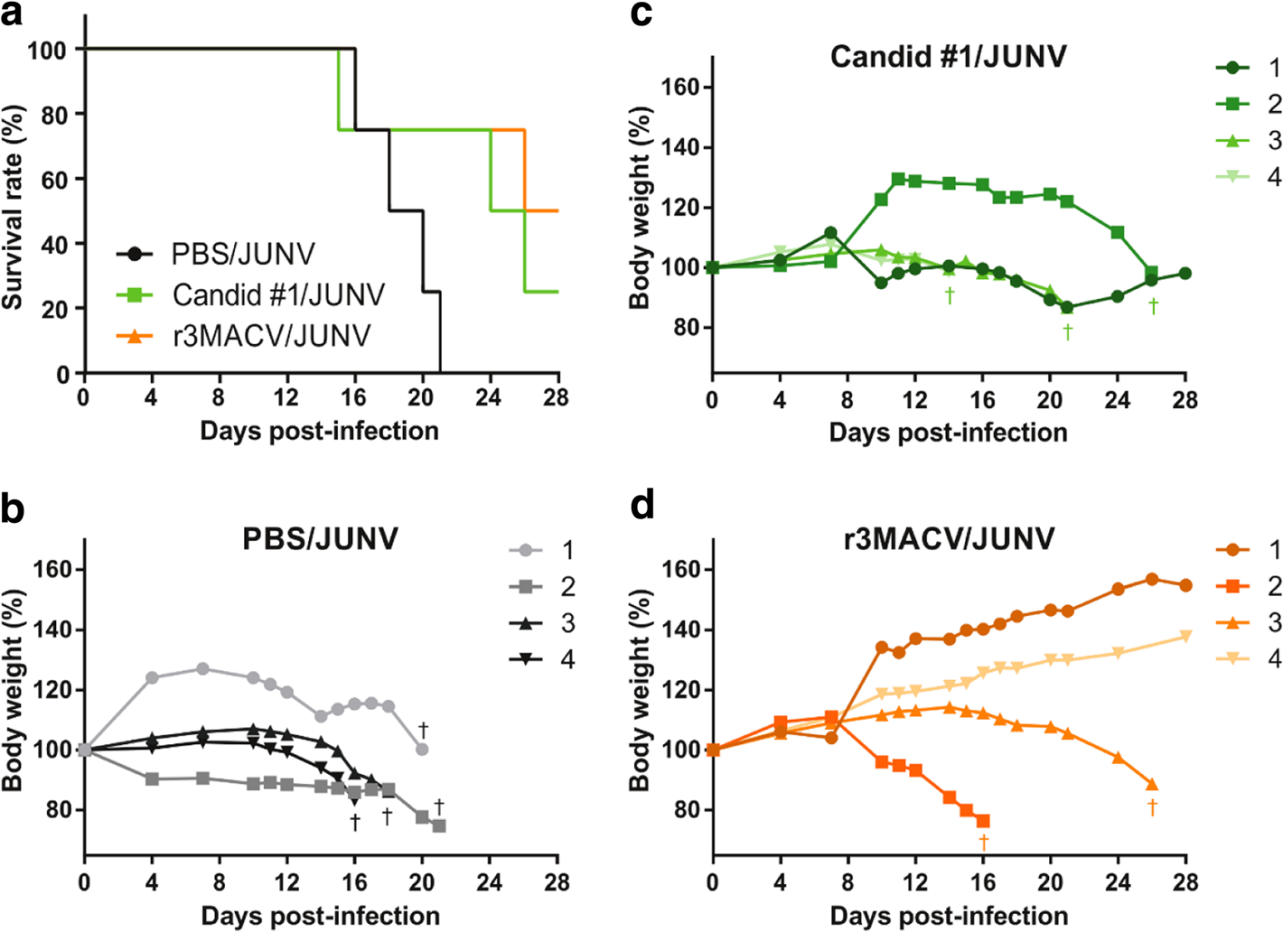
Survival rate and weight changes of guinea pigs throughout the experiment. Three groups of Hartley guinea pigs (*n* = 4/group) were immunized intraperitoneally in the right flank with 3 × 10^4^ TCID_50_ of Candid #1, 4 × 10^3^ TCID_50_ of r3MACV or PBS (control). Simultaneously, in the left flank, a lethal challenge (10^4^ TCID_50_ of JUNV strain Espindola) was injected intraperitoneally. **a** Their survival rate and **b**-**d** weight were observed for 28 days thereafter. The † indicated animals that either succumbed to the infection or were humanly euthanized before the end of the experiment

All control guinea pigs, receiving JUNV and PBS, developed severe symptoms before dying including high weight loss, dyspnea and decrease of tonus (Fig. 4a and b). Similar symptoms and outcomes were observed in all the non-protected animals in other group (Fig. 4a). Animals developing a fatal infection lost from 9 to 34% of their maximum weight (Fig. 4b, c and d). Surviving animals, either in the Candid #1/JUNV group (one, 25%) or in the r3MACV/JUNV group (two, 50%), did not show any symptoms. The Candid #1/JUNV group survivor, guinea pig #1, lost up to 12.8% of its maximum weight from day 16 pi to day 21 pi, then regained 98.5% of its weight (Fig. 4c). The surviving guinea pigs #1 and #4 from the r3MACV/JUNV group, regularly kept gaining weight, reaching at the end of the experiment 155 and 138% of their initial weight, respectively (Fig. 4d).

**Fig. 5:**
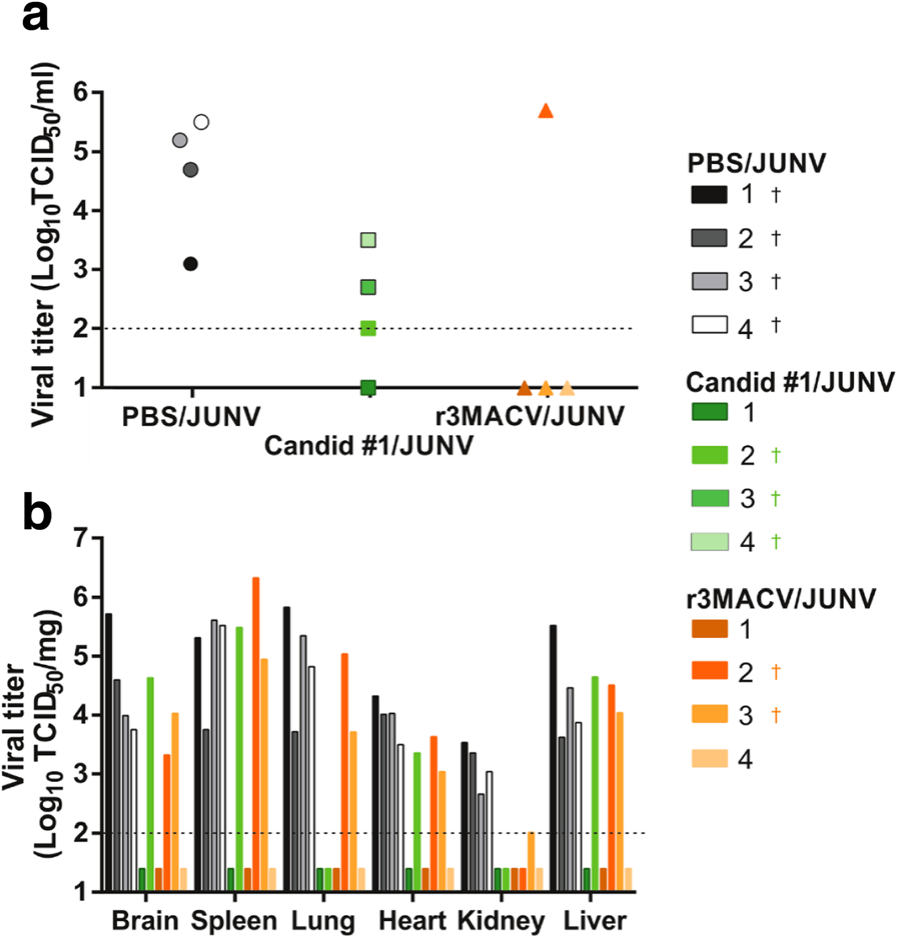
JUNV viremia and viral titer in organs of guinea pig in the three conditions. **a** Blood samples were collected at 14 days post-infection in the retro-orbital sinus. Blood samples were centrifuged (1400 rpm for 3 min) to collect sera. Virus titers were determined, with a limit of detection at 10^2^ TCID_50_/ml (dotted line). **b** Following the euthanasia of animals, organs (brain, spleen, lung, left kidney, heart and liver) were collected and stored at − 80 °C. Organs of animals #3 and #4 of the C #1/JUNV group were not collected because they were found dead with cadaveric rigidity. Collected organs were cut, weighted and crushed using Tissue Lyser in 400 μl PBS (ThermoFisher Scientific). Supernatant solutions were harvested and used for virus titration, with a limit of detection at 10^2^ TCID_50_/mg (dotted line). The † indicated animals that either succumbed to the infection or were humanly euthanized before the end of the experiment

All guinea pigs which succumbed to the infection showed a detectable JUNV viral load in their serum at day 14, except guinea pig #3 from the r3MACV/JUNV group, which died at 26 dpi (Fig. 5a). We observed that when the guinea pig JUNV viremia was above 10^2^ TCID_50_/ml at day 14, the guinea pig died between 14 and 21 dpi. None of the surviving animals exhibited any detectable viremia at day 14. All dead animals had detectable virus titers in all tested organs except in the lung of animal #2 of the Candid #1/JUNV group, and in the kidney of animal #2 of the r3MACV/JUNV group. All surviving animals did not exhibit any detectable viral titer in tested organs (Fig. 5b).

In conclusion, our study demonstrated that the r3MACV candidate was able to protect 50% of guinea pigs from a simultaneous lethal JUNV challenge. Surviving animal displayed neither clinical symptom nor detectable infectious virus in collected samples. This protection was higher as a therapeutic treatment than that provided by Candid #1 (as a vaccine requiring two months for immunity with variable efficacy against different JUNV strains [14]). Moreover protection occurred at a 3-fold lower dose of r3MACVthan Candid #1. Several factors may be considered: (i) as observed in vitro, the r3MACV might have induced a high level of type-I IFN production, resulting in the establishment of an antiviral state preventing the JUNV propagation, ultimately resulting in the control of the viral infection by the host immune system; (ii) the r3MACV might have competed with JUNV to enter cells, or induced the superinfection exclusion phenomenon [23], thereby limiting the ability of the pathogenic JUNV to infect animals; (iii) the r3MACV might have also elicited an adaptive immune response. These hypotheses will need to be tested in future experiments.

We could not test the attenuation of the r3MACV in a MACV wt lethal challenge as noted (Additional file 1) and it could be studied if such a successful animal model is identified. However, these studies suggest thatr3MACV will be a safe product because (i) the tri-segments modification of the mammarenavirus genome was associated with an attenuated phenotype [10], (ii) the NP D380A mutation was shown to block the inhibition of the IFN-1β production in A549 cells, (iii) the 2-log reduction ability of the r3MACV to grow in VERO cells, (iv) a MACV and JUNV recombination viruses could not be excluded but likely improbable [23].

Regarding the efficacy of Candid #1 as a therapeutic vaccine, we showed that it was less protective than the r3MACV despite its closer antigenic relationship with the virus used for the challenge. If the r3MACV fulfills the necessary requirements in term of safety and antigenic expression of GPCΔ33 inserted in each S segment, further experiments will be needed to address its potentially therapeutic and prophylactic protection against MACV and JUNV and ultimately against other South America arenaviruses responsible for HHF.

## Additional files

Additional file 1: Impact of the mutation L166G on the viral polymerase main functions. BHK-21 cells were transfected with plasmids expressing the minigenome (MG), the MACV nucleoprotein (NP) and the wild-type AY619642 (L) or the 166G-mutated (E166G) polymerase in conditions previously published [11]. The plasmid expressing the MACV polymerase was replaced by a plasmid expressing the mCherry in the negative control. Three days post-transfection, the *Gaussia* Luciferase expression in the supernatant solution was evaluated using the *Gaussia* luciferase assay kit (New England Biolabs). Asterisks denote significant differences with control (*P* < 0.05, one-way ANOVA with the Bonferroni correction, **** ≤ 0.0001). (PDF 313 kb)

Additional file 2: Genomic sequences of r3MACV. (PDF 336 kb)

Additional file 3: Survival rate and body weight of guinea pig inoculated with two doses of wt MACV. Four or five guinea pigs were infected intraperitoneally with 3 × 10^4^ TCID_50_ (low dose) or 10^6^ TCID_50_ (high dose) of wt MACV, respectively. The animals were observed for 24 days thereafter. (A) Their survival rate and (B) their weight were monitored. Animals infected with the high dose were heavier (515-610 g) than the ones used for the low dose experiment (250-300 g), explaining the differences of weight evolution between the two groups. (PDF 375 kb)

## Abbreviations

BHF: Bolivian Hemorrhagic Fever
BSL: Biosafety Level
CHAPV: Chapare virus
GPC: Glycoprotein complex
GTOV: Guanarito virus
HHF: Human Hemorrhagic Fever
hpi: Hours post-infection
IFN: Interferon
JUNV: Junín virus
LCMV: Lymphocytic choriomeningitis virus
MACV: Machupo virus
MOI: Multiplicity of infection
NHPs: Non-human primates
NP: Nucleoprotein
SABV: Sabiá virus
wt: Wild-type
