# Supplementary figures and images for: Description and characterization of a novel live-attenuated tri-segmented Machupo virus in Guinea pigs
Source: Virol J. 2018 Jun 7;15:99. doi: 10.1186/s12985-018-1009-4 (PMC5992841; doi:10.1186/s12985-018-1009-4)

**SUPPLEMENT FILE 1**

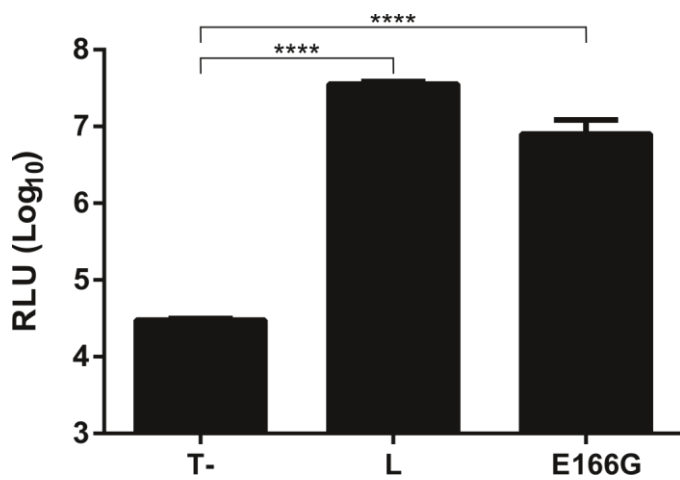

Supplement: Supplementary file 1 — Impact of the mutation L166G on the viral polymerase main functions. BHK-21 cells were transfected with plasmids expressing the minigenome (MG), the MACV nucleoprotein (NP) and the wild-type AY619642 (L) or the 166G-mutated (E166G) polymerase in conditions previously published [11]. The plasmid expressing the MACV polymerase was replaced by a plasmid expressing the mCherry in the negative control. Three days post-transfection, the Gaussia Luciferase expression in the supernatant solution was evaluated using the Gaussia luciferase assay kit (New England Biolabs). Asterisks denote significant differences with control (P < 0.05, one-way ANOVA with the Bonferroni correction, **** ≤ 0.0001). (PDF 313 kb) [file 12985_2018_1009_MOESM1_ESM.pdf]

**SUPPLEMENT FILE 3**

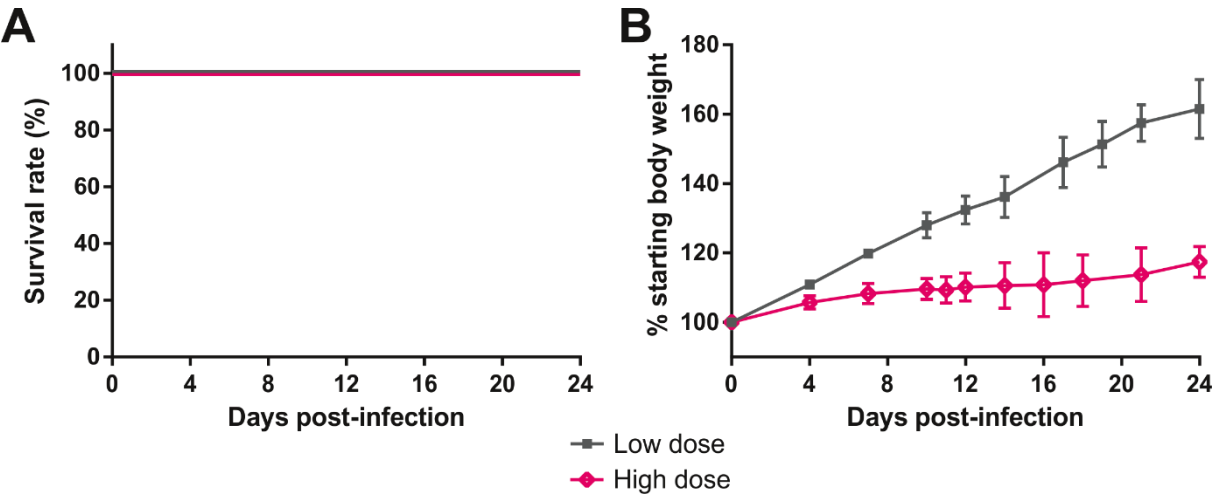

Supplement: Supplementary file 3 — Survival rate and body weight of guinea pig inoculated with two doses of wt MACV. Four or five guinea pigs were infected intraperitoneally with 3 × 10^4^ TCID50 (low dose) or 106 TCID50 (high dose) of wt MACV, respectively. The animals were observed for 24 days thereafter. (A) Their survival rate and (B) their weight were monitored. Animals infected with the high dose were heavier (515-610 g) than the ones used for the low dose experiment (250-300 g), explaining the differences of weight evolution between the two groups. (PDF 375 kb) [file 12985_2018_1009_MOESM3_ESM.pdf]
