# Supplementary material for: Description and characterization of a novel live-attenuated tri-segmented Machupo virus in Guinea pigs
Source: Virol J. 2018 Jun 7;15:99. doi: 10.1186/s12985-018-1009-4 (PMC5992841; doi:10.1186/s12985-018-1009-4)
Supplement: Supplementary file 2 — Genomic sequences of r3MACV. (PDF 336 kb) [file 12985_2018_1009_MOESM2_ESM.pdf]

SUPPLEMENT FILE 2

NP sequence of r3MACV

ATGGCTCACTCCAAGGAAATTCCCAGCTTTCGGTGGACTCAGTCACTGAGAAGAGGCCTGAGTCAGTTCACCC
ACACTGTGAAAACAGATGTGTTGAAAGATGCCAAGCTCATAGCTGACAGCATCGACTTCAACCAGGTTTCACA
AGTGCAGAGGGCTCTCAGAAAGAACAAAAGGGGTGAAGAGGATCTGAACAAGCTGAGGGATTTAAACAAAG
AAGTGGATAGGCTCATGTCTATGAAAAGCATCCAGAAAAACACCATATTCAAGATTGGAGATCTGGGGAGAG
ATGAATTGATGGAGCTTGCATCAGACTTGGAAAAACTGAAGAACAAGATAAAGAGGACTGAGTCAGGTCCCC
AAGGGCTGTACATGGGTAATTTGTCACAGCTGCAACTGACAAAGAGGTCAGAAATCTTAAAAACCCTGGGATT
CCAACAGCAGAGAGGTGCTGGAAATGGTGTGGTTAGAATCTGGGATGTCTCAGATCCATCAAAACTGAATAAT
CAGTTTGGTTCCATGCCAGCTCTCACAATCGCTTGTATGACTGTCCAGGGTGGAGAAACGATGAATAGTGTGG
TCCAAGCGTTGACATCTCTGGGCCTGTTATACACTGTTAAATATCCGAATTTAAATGACCTTGACAAGCTAACTC
TAGAGCACGAATGCTTGCAGATCGTAACTAAGGACGAGAGCTCCATCAACATCTCTGGCTATAACTTCAGTCTG
TCAGCTGCTGTGAAAGCTGGCGCCTCAATACTTGACGGTGGGAACATGCTGGAAACAATTAGGGTCACTCCTG
ATAATTTCTCCAGCTTGATTAAATCAACTCTGCAAGTCAAGCGAAAAGAGGGGATGTTTATAGACGAGAAACC
TGGGAATCGAAATCCTTATGAGAACCTTCTGTATAAATTGTGTCTCTCAGGTGACGGGTGGCCTTACATTGGTT
CCAGATCACAAATTCTTGGGAGATCTTGGGACAACACAAGTGTTGATCTAACAAAGAAACCTCAAGTTGGACC
GAGACAACCCGAGAAAAACGGTCAGAATCTAAGACTAGCAAACCTGACTGAAATGCAAGAAGCAGTGATTAA
AGAGGCTGTAAAGAAGTTAGACCCCACTAATACACTGTGGCTTGCCATTGAAGGGCCTCCAACAGACCCTGTG
GAATTGGCACTATATCAGCCAGCCAACAAGCATTATATTCATTGTTTTAGAAAGCCACATGATGAGAAGGGCTT
CAAAAATGGCAGCAGACATTCACATGGCATCTTGATGCAAGACATCGAGGATGCAATGCCAGGAGTATTAAGT
TATGTAATAGGTTTACTACCACAAGATATGGTGATTACAACTCAAGGTTCTGACGACATAAGGAAACTTTTAGA
CATTCATGGACGGAAGGATTTAAAGCTGGTAGATGTGAAACTCACATCTGATCAAGCAAGACTCTATGATCAG
CAAATTTGGGAGAAGTTTGGACATCTTTGCAAACATCATAATGGAGTTGTTGTCAACAAGAAAAAGAGAGAAA
AAGACTCTCCATTCAAATTGAGTTCTGGTGAACCTCACTGTGCTCTGTTGGATTGTATCATGTATCAATCAGTGA
TGGATGGCAAAATGGTAGATGAAGAACCAGTGGCACTTTTACCTCTCAGCCTTCTATTTCTACCCAAGGCAGCC
TTTGCACTGTAA

GPC sequence of r3MACV

ATGGGGCAGCTTATCAGCTTCTTTCAGGAGATTCCTGTTTTTCTACAGGAAGCTCTGAACATCGCTTTAGTGGCT
GTTAGTCTCATAGCTGTCATCAAAGGCATCATTAACCTTTACAAAAGTGGTCTCTTCCAGTTCATCTTCTTTCTCC
TCCTAGCAGGGAGGTCCTGCTCGGATGGCACATTCAAAATAGGCCTACACACTGAGTTCCAGTCAGTCACCCTT
ACCATGCAGAGACTTTTAGCTAACCATTCAAATGAGCTCCCATCTCTCTGCATGCTTAACAATAGTTTTTATTAT
ATGAGGGGAGGTGTGAACACCTTCCTGATTCGTGTTTCTGATATTTCAGTCCTCATGAAGGAGTATGATGTATC
AATCTATGAACCAGAGGATCTTGGAAATTGTCTTAACAAGTCTGACTCAAGCTGGGCTATTCATTGGTTCTCAA
ATGCTTTGGGACATGACTGGCTTATGGATCCTCCAATGCTATGTAGAAACAAGACAAAGAAGGAGGGATCTAA
CATTCAATTCAACATCAGCAAAGCTGATGATGCCAGAGTGTATGGAAAGAAGATAAGAAATGGTATGAGGCA
TCTCTTCAGGGGCTTCCATGACCCGTGTGAGGAAGGGAAAGTGTGCTACCTGACCATCAATCAGTGTGGTGAC
CCCAGTTCCTTTGACTACTGTGGCGTGAATCATCTTTCCAAATGTCAGTTTGACCATGTGAACACCCTTCATTTCC
TTGTGAGAAGTAAGACACATCTCAACTTTGAGAGGTCTTTGAAAGCATTTTTCTCATGGTCTCTGACAGACTCCT
CAGGAAAGGACATGCCAGGAGGTTATTGTCTAGAGGAATGGATGTTGATAGCAGCCAAAATGAAATGTTTCG
GAAACACTGCTGTTGCTAAATGTAATCAAAATCATGACTCAGAGTTCTGTGATATGCTGAGGCTATTCGACTAT
AACAAGAATGCAATAAAGACCCTCAATGATGAATCAAAGAAAGAAATCAATCTTCTAAGCCAGACAGTGAATG
CCTTAATCTCAGATAATTTGTTAATGAAGAATAAAATTAAAGAGCTAATGAGCATCCCTTATTGTAATTACACAA
AGTTTTGGTATGTCAATCATACCCTGACAGGGCAGCACACTCTTCCAAGATGTTGGTTGATAAGGAATGGAAG
TTATCTTAACACTTCTGAATTCAGGAATGACTGGATTTTAGAGAGTGATCACCTCATCTCAGAGATGTTAAGTA

AGGAATATGCTGAAAGGCAAGGCAAAACCCCAATCACATTAGTTGATATTTGTTTCTGGAGCACAATTTTCTTCACAGCATCATTGTTCCTTCATCTAGTCGGAATACCCACCCATCGACACCTCAAAGGCGAAGCCTGTCCTTTGCCTCATAAGCTGGACAGCTTCGGAGGTTGTAGATGTGGCAAATATCCCAGATTGAAGAAACCCACCATCTGGCACAAAAGACATTAA

L sequence of r3MACV

ATGGATGAATATGTCCAAGAGTTGAAAGGGCTTATTAGAAAGCACATTCCTGAGAGGTGCGAGTTTGGACATCAAAAGGTCACATTCCTATCTCAGGTGCATCCCAGTCCCTTGCTCACAGAAGGCTTCAAGTTGCTCTCTAGTCTTGTTGAACTTGAGTCCTGTGAAGCACACGCCTGCCAGGCAAACACTGATCAAAGATTCGTAGATGTAATACTATCTGACAATGGGATTTTATGTCCAACTTTACCAAAGGTGATCCCCGATGGGTTCAAATTAACTGGGAAAACCCTAATTCTGCTGGAGACCTTTGTAAGAGTAAACCCAGATGAGTTCGAGAAGAAATGGAAAGCTGACATGTCAAAGCTTTTAAACCTGAAGCATGACCTGCAAAAGTCGGGCGTGACTTTGGTTCCTATTGTTGATGGCAGGAGCAATTACAACAATAGATTTGTTGCTGATTGGGTGATTGAAAGAATAAGATGGTTGCTTATCGAGATTTTGAAAGCATCCAAATCCATGCTTGAGATTGATATTGAGGACCAGGAATATCAACGATTAATTCATAGTCTATCAAACGTTAAGAATCAAAGCTTGGGGTTGGAAAACCTAGAACATTTGAAAAGGAATTCTTTAGATTATGATGAGAGGTTGAATGAGTCATTGTTTATTGGTTTGAAAGGAGACATTAGAGAGTCGACAGTGAGGGAAGAACTCATCAAACTTAAATTGTGGTTCAAGGATGAGGTATTCAGCAAAGGTCTGGGCAAATTCAAATTGACTGACAGAAGGGAATTATTAGAGTCCCTATCGAGCTTAGGTGCTCATCTAGATAGTGATGTGTCAAGCTGTCCCTTTTGTAACAATAAGTTGATGGAAATTGTTTACAATGTGACCTTTAGCTGTGTTGAAAGGACCGATGGTGTAGCCACAGTGGACCAGCAGTTTAGCACCACACACTCTAACATAGAAAAACATTACCTTAGTGTCTTATCTTTGTGCAATAAAATAAAGGGGTTGAAGGTCTTTAACACCAGAAGAAACACCCTACTATTCTTAGATCTCATAATGGTTAATTTGATGGTTGACATTAGTGACAGTTGCCAGGACGCGATTGAAAGTTTGAGAAAATCGGGATTGATAGTGGGTCAGATGGTTATGTTAGTGAATGATAGAGTGCTGGACATTTTGGAGGCTGTTAAACTCATCAGAAAAAAGATTGGGACTAATCCCAATTGGGTCAAGAATTGTTCAAAAAATTCTAGAGAGATCACATCCAGAAATATGGCATCACTTAAGCACTTTGATAAAGCAACCAGACTTCAATAGCTTGATTTCAATAGCCCAACATCTTGTCTCAGATAGACCAATTATGAGGTATTCAGTGGAAAGAGGCTCTGATAAGATTTGTAGACATAAATTGTTCCAGGAGATGTCTAGCTTTGAGCAAATGAGGTTATTCAAGACTTTGTCCTCAATATCCTTGTCACTAATCAATTCTATGAAAACATCCTTTTCCTCGAGATTGCTTGTGAATGAGAGAGAATTTTCTAAGTACTTTGGTAATGTTAGACTCAGAGAGTGTTATGCCCAAAGGTTTTATTTGGCTGAGAGTTTAGTTGGCTTTCTTTTCTATCAAAAAACAGGTGAAAGGTCCAGGTGTTATTCAGTTTATTTATCTGATAACGGTGTGATGTCAGAACAGGGTTCATTTTATTGTGATCCAAAGAGATTCTTCTTGCCTGTTTTTTCTGATGAAGTTTTGGCTGGAATGTGTGAGGAAATGACAAGCTGGCTGGATTTTGACACCGGATTAATGAATGACACTGGGCCCATATTAAGGTTGTTAGTTCTGGCTATACTCTGCTCCCCATCAAAGAGGAATCAAACCTTTCTACAAGGATTAAGATACTTTTTAATGGCTTTTGCTAACCAAATTCATCATATCGACCTGACATCTAAATTGGTAGTGGAATGCAAGTCAAGTTCAGAAGTGGTGGTTCAAAGGCTTGCAGTGGGACTCTTCATTAGACTACTTAGTGGTGAGTCAGATGCAAGTTTATTTTTCTCAAGAAGATTCAAATATCTGCTAAATGTTAGTTACCTTTGCCATTTAATCACAAAAGAAACGCCTGACCGATTAACAGATCAGATCAAGTGTTTTGAGAAGTTCATTGAGCCCAAAGTTAAGTTTGGATGTGCTGTGGTGAACCCATCTCTAAATGGAAAGCTGACTGTAGATCAGGAGGATATTATGATAAATGGTTTAAAGAAGTTTTTTTCAAAATCCTTAAGGGACACTGAGGATGTGCAGACACCAGGTGTGTGTAAAGAACTCTTAAATTATTGTGTGTCTTTGTTTAATAGGGGAAAGTTAAAGGTTTCTGGGGAGCTTAAAAATAATCCCTTTAGACCTAATATAACAAGTACTGCTTTGGACTTATCAAGTAACAAGAGTGTTGTTATACCAAAACTAGATGAGTTAGGCAACATCCTCTCCACCTATGACAAGGAAAAACTAGTGTCAGCATGTGTTTCCTCTATGGCAGAGAGGTTTAAAACCAAAGGAAGGTACAATCTAGATCCAGATTCAACTGACTATTTGATTCTAAAAAATCTGACAGGTTTGGTGAGTGCAGGGCCGAAAGCTAAGAGTACTCAAGAAGAATTGTCTTTAATGTACGAGGCGTTAACAGAAGAACAGGTTGAATCATTCAACGAAATTAAACATGATGTACAAGTTGCCTTAGCCAAGATGGCTGACAACAGTGTGAACACAAGAACTAAAAATCTCGGCCGAGCTGATAATTCAGTAAAAAATGGGAATAATCCTTTGGACAATTTGTGGTCACCTTTTGGGGTGATGAAAGAGATT

92 AGAGCTGAAGTATCGTTACACGAAGTCAAGGATTTTGACCCAGATGTTCTTCCTCCTGAGGTGTACAAGGAGT
93 TGTGTGACGCAGTTTACAAATCGTCAGAAAAATGCAACTTCTTCCTTGAGGGAGTACTTGATGTCTGTCCCTTG
94 GGGTTGCTGCTGAAAAATCTGACCACATCAAGTTATGTGGACGAAGAGTATTTTATGTGTTTTAAATACCTCTT
95 GATACAAGGTCACTTTGATCAAAAACTAGGAAGTTATGAGCACAAAAGCAGATCTAGACTGGGTTTCACCGAT
96 GAGACATTAAGACTCAAAGACGAGGTTAGACTTAGTATAAGAGAAAGCAACTCAGAGGCGATTGCAGACAAG
97 TTGGATAAAAGTTACTTCACCAATGCCGCATTAAGGAATCTGTGTTTTTATTCTGAAGATTCGCCCACTGAGTTC
98 ACAAGCATTAGTTCCAACTCAGGAAACCTTAAATTTGGTCTAAGTTATAAGGAACAGGTGGGTTCAAACAGAG
99 AGCTCTATGTAGGAGACCTCAACACAAAACTGATGACAAGATTGGTGGAGGATTTTTCTGAAGCTGTAGGGAA
100 TTCTATGAAGTACACCTGTCTGAACTCTGAAAAAGAATTTGAACGGGCCATTTGTGACATGAAGATGGCAGTG
101 AATAATGGTGATTTGTCATGTTCTTATGATCACTCAAAATGGGGTCCAACAATGAGTCCTGCACTTTTCCTGGCA
102 TTATTACAAATGCTGGAATTGAGAACCCCTGTTGACAGGTCAAAAATTGATCTTGATTCTGTTAAAAGTATACT
103 GAAATGGCACCTTCACAAAGTTGTAGAAGTGCCTATCAATGTGGCTGAAGCCTACTGTATTGGAAAGCTAAAA
104 AGGTCCCTTGGATTGATGGGCTGCGGTTCCACCAGTTTGAGTGAAGAATTCTTCCATCAGACCATGCAGTTAAA
105 TGGTCAGATTCCTAGTCACATTATGTCTGTTTTGGACATGGGGCAAGGGATACTACATAACACTTCTGATTTGT
106 ATGGATTAATCACAGAGCAATTTTTGTGTTATGCACTCGATCTTTTGTATGATGTAATCCCTGTGTCATATACCT
107 CTAGTGACGATCAGATCACTCTTATCAAGACACCTTCTCTGGATATAGAAGGGGGTTCAGATGCTGCGGAGTG
108 GCTAGAAATGATCTGCTTCCATGAGTTTCTGTCATCTAAATTGAATAAGTTTGTCAGTCCCAAGAGTGTCATCG
109 GGACTTTTGTCGCAGAGTTCAAATCAAGATTTTTTGTCATGGGTGAAGAAACACCATTGCTCACAAAGTTTGTT
110 GCTGCAGCTTTACACAATGTAAAGTGTAAAACACCCACACAATTGTCAGAAACTATAGATACCATCTGTGATCA
111 ATGCATTGCAAATGGAGTCAGCACCAAAATAGTGACAAGAATATCTAAGCGAGTTAACCAATTAATTAGATAC
112 TCCGGATATGGAGAGACCCCCTTTGGGGCTATTGAAGATCAAGATGTGAAAGACTGGGTGGATGGCTCAAGG
113 GGTTACCGTCTACAGAGAAAGATTGAAGCTATCTTTCATGATGACAAGGAAACCTCTTTTATTAGGAACTGTGC
114 CAGAAAGGTTTTTAATGATATAAAGCGTGGCAGAATATTTGAGGAGAATCTGATCAATTTGATTGGTAGAGGT
115 GGTGATGAGGCCTTGACTGGTTTTTTACAGTATGCAGGTTGCAGTGAACAAGAAGTTAATCGCGTGTTGAACT
116 ATAGATGGGTGAATTTATCAAGTTTTGGAGATCTTAGGTTAGTTTTGAGGACAAAGTTGATGACAAGTAGAAG
117 AGTTCTTGAAAGGGAGGAAGTGCCAACTTTAATTAAAACCTTGCAATCCAAACTTTCCAGAAACTTCACAAAAG
118 GAGTTAAGAAGATCCTTGCTGAGTCAATTAATAAGTCAGCTTTCCAGTCATCCGTTGCTAGCGGTTTTATTGGG
119 TTCTGCAAAAGCATGGGTAGCAAGTGCGTTAGAGATGGGAAAGGGGGATTCTTATACATAAAAGAAGTGTAT
120 TCAGGTGTAAGTGCTTGTACATGTGAAATATGTGCGTTAAAGCCCAAGATCATATATTGCAACAACTCACTTAA
121 CAAAGTGAGTCAGTTTTCAAAACCTATTTTGTGGGATTATTTTTCATTAGTCCTAACAAATGCATGTGAACTTGG
122 AGAGTGGGTGTTTTCCACAGTTAAAGAGCCCCAAAAGCCATTGGTGTTGAACAACCAAAACTTTTTTTGGGCTG
123 TGAAGCCTAAAGTGGTGAGACAAATTGAGGATCAACTGGGAATGAACCATGTTCTGCAATCTATAAGGAGAA
124 ATTATCCAGTTCTTTTTGACGAACACTTGACTCCATTTATGAATGATCTTCAGGTCAGCAGAACAATGGATAGC
125 GGGAGGCTAAAATTTCTGGATGTCTGCATTGCTTTAGACATGATGAATGAAAACCTTGGTATCATCAGTCACCT
126 GTTAAAAACCAGAGACAACAGTGTTTACATAGTTAAACAAAGTGATTGTGCCTTAGCACACATAAGGCAGTCA
127 AGCTACACAGATTGGGAATTAGGACTTAGTCCGCAGCAGATTTGCACAAACTTCAAGACCCAGCTGGTCCTAT
128 CTTCAATGGTAAACCCATTAGTCCTTTCAACATCTTGTCTGAAGTCATTTTTCTGGTTCAATGAAGTCCTAGAGC
129 TAGAGGATGATAGTCAAATTGAGTTAGCAGAATTGACTGACTTTGCCTTAATGGTTAAGAATCAAAATGTTTCC
130 AGAGCTATGTTTGTGGAGGATATCGCAATGGGGTATGTTGTCTCTAACTTCGAGGGTGTTAGAATCTCGTTAA
131 GCAACGTGATGGTTGATGGTGTTCAATTGCCGCCTCAGGAGAAGGCTCCTGATATAGGTGAATTGTTTGGATT
132 GAAGGCTGAGAATGTCATTGTGGGCCTAGTGGTGCAGATCGATCATGTAAGAATGTCAACGAAGTTCAAGTT
133 GAAGAGAAAAATGGTGTATAGTTTCTCTTTGGAATGCATAATGGATGTAGGAGAGATCCAGAACAAAGAGGT
134 TATTTTGAAGGTGGTGGCAGTCGATCAGTCTGTGTCTGGGTCAGGAGGAAACCATATGCTGCTGGATGGTGTG
135 TCGGTTGTTGCAAGCCTCCCATTATTTACTGGGCAGGCCAGTTTTGATTTGGCTGCAATGTTAATCGAGTCAAA
136 TCTGGCTGGCTCAAATGATAATTTCTTAATGAGAAATGTTACCTTGGATTTAGGTGGATTTTCCCCAGAGTTGA
137 GTGACAAATATTCCTACAGGCTCTCGGGACCTGAAAACCAAGAGGATCCGTTGGTCTTGAAGGATGGTGCCTT
138 CTATGTGGGAGGTGAGAGACTGTCCACATATAAAGTAGAGTTTACAGGTGATCTTGTGGTGAAAGCATTGGG
139 GGCCCTAGAAGATGATGAGAGTGTTGTTTCTATGCTGCACCAACTGTGGCCTTATTTGAAAGCAACATCACAG

GTTATTTTGTTTCAACAGGAAGATTTCACCATTGTTCATGATTTGTACAAGAAACAGCTCACAAAATCAATTGAAAGCTTTGGCGAATGGATCGAATTTACCAACTTTAAGGTTGCCTATAGTAAATCACTCAAAGAGCTTGTGATATCAGACACCCAGGGGTCATTCAGACTGAAGGGTGTGATGTGTAGACCCCTGGCAAGCACGCCCCAAGTGGAGGACATTGAGTAA

Z sequence of r3MACV

ATGGGCAACTGCAATAAGCCTCCCAAGAGGCCTCCCAACACACAAACATCATCCAACCAACCCTCAGCAGAGTTCAGGAGAACAGCCCCACCCAGTCTCTATGGTCGCTACAACTGCAAATGCTGTTGGTTTGCCGACACAAACTTGATTACATGTAACGACCACTACTTGTGCCTGAGATGTCATCAAACAATGCTCAGGAATTCTGAACTCTGTCACATATGCTGGAAACCACTACCGACATCCATCACAGTTCCCGTGGAGCCAAGTGCCCCCCCACCATAA

GPCΔ33 GTOV sequence

ATGGGACAGTTAATCAGTTTCTTTCAGGACATACCGATCTTCTTTGAAGAAGCACTCAATGTAGCCTTGGCTGTGGTTACACTGCTTGCTATTATAAAGGGCATTGTGAATGTTTGGAAATCAGGAATTCTTCAGCTGTTTGTGTTCTTGGTTCTGGCGGGAAGGTCATGCTCTTTCAAAGTTGGTCATCATACAAATTTTGAGTCATTTACGGTTAAGCTAGGAGGTGTTTTTCATGAATTACCTTCACTATGTAGGGTCAACAACTCTTACAGTCTAATTAGGCTTTCTCATAACAGTAATCAGGCATTATCAGTTGAGTATGTGGATGTGCACCCTGTCCTCTGTTCGTCCAGTCCAACCATACTCGACAACTACACTCAATGTATAAAAGGCTCTCCAGAGTTTGATTGGATTCTTGGGTGGACAATTAAGGGATTGGGACATGACTTTTTGAGGGATCCAAGAATCTGCTGTGAGCCTAAAAAGACAACTAATGCTGAATTTACATTCCAATTGAATTTAACAGATAGTCCTGAGACCCATCACTATAGGAGCAAGATTGAGGTAGGCATCCGACATTTATTCGGGAACTACATAACTAATGATAGCTATTCAAAAAATGTCTGTGGTTATGAGGAACACTACCTGGGAAGGTCAATGCTCAAATAGTCATGTGAATACACTGAGATTTCTTGTTAAAAATGCAGGTTACCTTGTTGGAAGAAAACCACTGGCATTTTTTAGTTGGTCACTTTCTGACCCAAAGGGTAATGACATGCCAGGTGGTTACTGTCTTGAAAGGTGGATGTTGGTTGCTGGGGATTTGAAGTGCTTTGGCAACACAGCTGTCGCCAAGTGTAATTTGAATCATGATTCTGAGTTCTGTGACATGTTGAGGCTGTTTGATTTCAATAAGAATGCCATTGAAAAACTGAACAACCAAACTAAAACTGCTGTCAACATGTTGACTCACTCAATAAATAGTCTAATATCTGATAATTTATTGATGAGAAACAAACTGAAGGAGATTTTGAAGGTCCCATATTGCAACTACACAAGATTCTGGTACATAAACCACACGAAATCTGGTGAGCACTCACTGCCTCGGTGTTGGCTGGTCAGTAATGGTTCCTATTTGAATGAGAGTGATTTTAGAAATGAGTGGATCTTGGAGAGTGATCACCTAATAGCAGAGATGTTAAGCAAAGAATACCAAGATAGGCAGGGTAAAACTCCCCTAACACTGGTTGATCTATGTTTCTGGAGTGCAATCTTTTTCACCACAAGTCTCTTTTTACACCTGGTTGGGTTCCCAACACATAGACATATACAGGGT

GPCΔ33 CHAPV sequence

ATGGGTCAACTTGTGAGTTTCTTTCAGGAAATTCCTAACATCATACAGGAAGCAATCAACATTGCTCTAATAGCTGTCAGCCTCATTGCTATCCTAAAAGGGTTGGTCAATCTGTGGAAAAGTGGTTTGTTTCAACTCCTGGTCTTCCTTATTCTAGCTGGAAGGTCCTGCTCATTCAAAATTGGAAGATCCACAGAACTCCAAAACATCACAATAAACATGTTAAAGGTCTTTGAGGACCATCCTATTTCTTGCACAGTGAACAAAACTCTTTATTACATCCGTGAAAGTGAGAATGCAACATGGTGTGTAGAGATTGCTGCACTGGACATGTCTGTCTTGCTCTCGCCACATGATCCACGTGTAATGGGCAACCTATCAAACTGTGTTCACCCAGACATTAAACATAGATCAGAACTTCTAGGTCTATTGGAGTGGATTTTGAGAGCTCTGAAATATGATTTTCTAAATTATCCACCCCTCTTGTGTGAAAAAGTGACATCATCTGTCAATGAAACACGCATTCAGATAAATGTGAGCGATAGTGCAGGTTCTCATGATTTCAAAGAAACCATGCTCCAAAGGCTGGCCAT

ACTATTTGGCACAAAATTGATGTTTGATAAAACACCTAAGCAGTTCATAGTTATCAGAAATCAGACTTGGGTGAATCAGTGCAAGTCCAATCATGTCAACACGTTGCATTTGATGATGGCTAATGCTGGGCATGCTGTTAAATTAAGAAGATTGCAAGGGGTGTTCACTTGGACGATCACAGATGCTGCTGGCAACGACATGCCTGGAGGTTATTGTTTGGAAAGGTGGATGTTAGTCACATCTGACCTGAAATGTTTTGGCAACACTGCTCTTGCAAAGTGTAACTTAAATCATGACTCTGAGTTCTGTGACATGTTGAAACTGTTTGAATTTAACAAAAAAGCAATTGAGTCATTGAATGACAACACAAAGAACAAAGTCAATCTATTAACACATTCCATTAATGCTTTAATTTCTGACAATCTTTTGATGAAGAATAGATTAAAAGAGTTATTGGACACTCCCTACTGCAACTACACAAAATTCTGGTATGTCAACCACACCATCACAGGAGAGCATTCACTTCCACGCTGTTGGATGGTTAAAAACAACAGCTACCTCAATGAAAGTGAATTTAGAAATGATTGGATTCTTGAGAGTGACCACTTGTTGTCCGAAATGTTGAACAAAGAGTATTTTGACAGACAAGGGAAAACCCCAATAACACTTGTTGACATCTGCTTTTGGAGCACACTGTTCTTCACAACAACATTGTTTCTCCATCTAGTAGGCTTCCCAACTCACAGACACATTCAGGGA
